# Facial Cellulitis of Unusual Odontogenic Origin

**DOI:** 10.3390/reports7030050

**Published:** 2024-06-21

**Authors:** Alexandre Perez, Valerio Cimini, Vincent Lenoir, Tommaso Lombardi

**Affiliations:** 1 Unit of Oral Surgery and Implantology, Division of Oral and Maxillofacial Surgery, Department of Surgery, Geneva University Hospitals, Faculty of Medicine, University of Geneva, 1205 Geneva, Switzerland; valerio.cimini@hcuge.ch; 2 Division of Radiology, Diagnostic Department, Geneva University Hospitals, University of Geneva, 1205 Geneva, Switzerland; vincent.lenoir@hug.ch; 3 Unit of Oral Medicine and Oral Maxillofacial Pathology, Division of Oral and Maxillofacial Surgery, Department of Surgery, Geneva University Hospitals, Faculty of Medicine, University of Geneva, 1205 Geneva, Switzerland; tommaso.lombardi@unige.ch

**Keywords:** cellulitis, odontogenic infection, dental fracture, trauma

## Abstract

A healthy man in his 40s was referred to the Oral Surgery and Implantology Unit of Geneva University Hospital for diagnosis and management of facial swelling affecting the right side of his lower jaw. The patient’s history revealed that the patient had been hit by several punches to the face a few months earlier. To investigate the swelling, an intra-oral radiograph, an orthopantomographic radiograph, and computed tomography were performed, which revealed no fracture of the lower jaw but the presence of a partly impacted fractured wisdom tooth (third molar). This finding, together with the clinical status, indicated cellulitis most likely related to the presence of a fractured wisdom tooth. The decision was made to proceed with tooth extraction, and follow-up at 3 weeks showed good healing and complete resolution of facial swelling. This case highlights that odontogenic infection can also occur as a result of necrosis following the fracture of an impacted wisdom tooth.

## 1. Introduction

Facial cellulitis of odontogenic origin is a deep, acute, and diffuse inflammation of the subcutaneous tissues that spreads through the spaces between several anatomic regions to the tissue cells [1]. Cellulitis of odontogenic origin accounted for 54% of pediatric patients with facial odontogenic cellulitis requiring hospitalization [2] and 50% of all facial infections [3]. The two factors upon which the severity depends are as follows: the immunocompromised status of the patient, and the severe virulence of the causative agent [4]. The alveolar maxillary bone is the primary local barrier. Most of the time, the infection spreads to the underlying soft tissues through the periosteum, the anatomical disposition of muscles and aponeuroses [4,5]. Facial cellulite is of dental origin in between 56 and 95% of cases [6]. According to several studies, caries is the cause of more than 90% of dental problems [7,8,9]. The main causal teeth are mandibular molars [9,10]. The first mandibular molar is particularly exposed to caries and related complications because of its appearance on the arcade at an age when oral hygiene is not yet assimilated, as well as its morphology [4]. Dental necrosis is found in up to 84.6% of these cases [4]. Periodontal infection, mainly pericoronitis of the mandibular wisdom teeth but also endo-periodontal lesions, represents the second dental etiology in up to 15% of cases [9,10]. At the mandible, the wisdom teeth have their apices located mainly against the lingual bone wall and below the line of insertion of the mylohyoid muscle. The resulting infections can directly seed the cervical area.

Our case is unique in that facial cellulitis was the consequence of a fractured impacted wisdom tooth with pulpal necrosis caused by violent blows to the mandible.

The aim of this article is to discuss an odontogenic infection of atypical etiology and to describe this unusual case to increase awareness of such rare event and to help other clinicians who might encounter similar cases. An additional message to take away from this article is not to underestimate impacted wisdom teeth and to always consider the patient’s medical history.

## 2. Detailed Case Description

A man in his 40s and in good health was referred by the Division of Prison Health to the Oral Surgery and Implantology Unit for diagnosis and treatment of facial swelling that had been present for several months and had worsened a few days earlier.

On the day of consultation, extraoral clinical examination showed facial swelling, neck lymphadenopathy, mild trismus, and painful palpation of the right side of the face.

Intraoral examination showed edentulousness of the right side of the lower jaw and revealed painful palpation at the site of the impacted lower wisdom tooth; at the same time, a small crestal fistula was present.

About six months earlier, during a fight, the man had allegedly received several punches to the face, which resulted in the fracture of several mandibular teeth. After the event, the man said he had removed the root remnants of the broken teeth himself.

**Figure 1:**
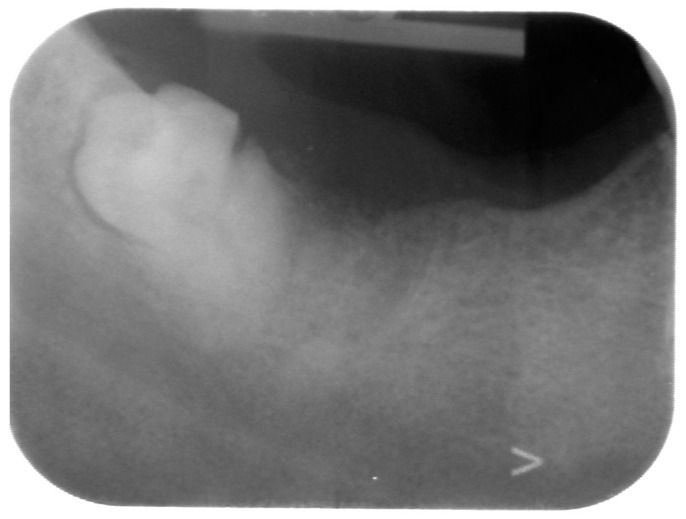
Intra oral X-ray showing a partly impacted fractured lower right wisdom tooth.

The first-level radiographic examination that was performed was an intra-oral radiograph, suspecting root remnants that were not removed following the punches (Figure 1). On RX, root remnants of teeth in lower right side were absent, but an impacted wisdom tooth was observed.

**Figure 2:**
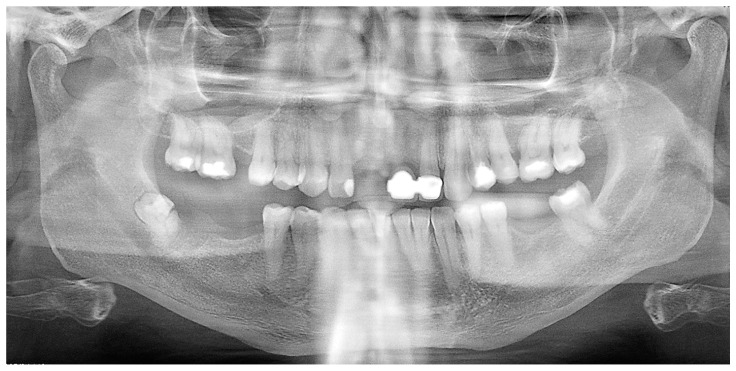
Orthopantomogram (OPT) confirming a semi-impacted fractured wisdom tooth on the right side of the jaw.

A further orthopantomogram X-ray was performed (Figure 2) and confirmed an impacted fractured wisdom tooth on the right side of the jaw.

**Figure 3:**
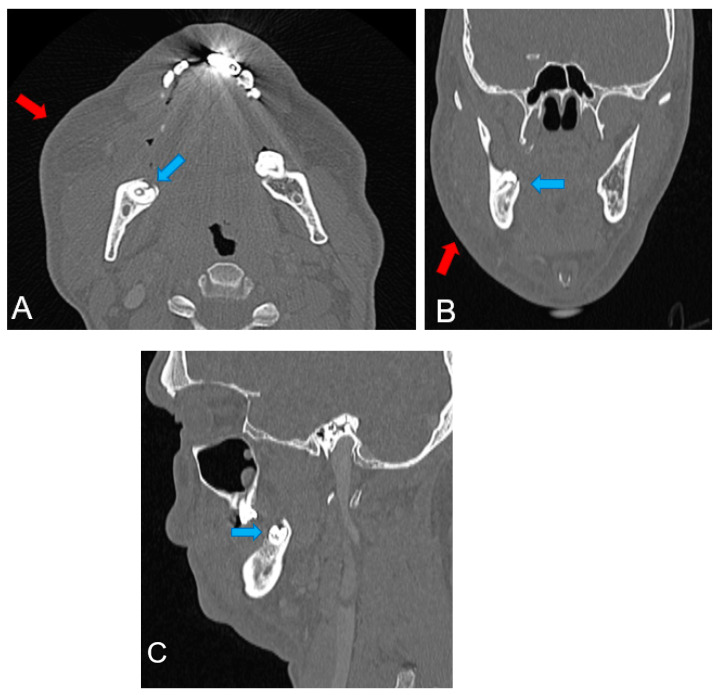
CT scan examination: (**A**) axial view; (**B**) frontal view; (**C**) sagittal view. The red arrow in (**A**,**B**) indicates the facial cellulitis lesion and the blue arrow in (**A**–**C**) the coronal fracture of the impacted lower right wisdom tooth.

To investigate a possible fracture of the mandible, a computed tomography (CT scan) examination was performed (Figure 3). It revealed right maxillary and mandibular subcutaneous swelling and infiltration, with thickening of the fascia and masseter muscle, without purulent collections. The CT scan revealed no fractures of the mandibular horizontal ramus. A diagnosis of cellulitis was made.

Regarding the treatment, following the appearance of swelling, the patient received Clavulanate-Amoxicillin therapy (Co-Amoxi-Mepha, Mepha Pharma, Aesch, Switzerland) (2 × 1 g daily) prescribed by the consultant of the Prison Health Unit 5 days previously, suspecting dermohypodermitis, which resulted in improvement of the swelling and trismus (two-finger trismus). The physician’s decision was to perform extraction of the tooth to remove the possible cause of the infection. The surgical wisdom tooth extraction was performed under local anesthesia (4% articaine with 1:100,000 adrenaline–Ubistesin™ Forte–3M ESPE, Stuttgart, Germany). An envelope flap was performed to expose the tooth, with an incision at the crestal level. The tooth was almost completely embedded in the bone; hence, a vestibular osteotomy and root separation were carried out to ease the removal. Co-amoxicillin (Co-Amoxi-Mepha, Mepha Pharma, Aesch, Switzerland) was continued for 6 days after surgery (2 × 1 g daily).

The 1-week follow-up showed good healing and complete resolution of the patient’s symptoms and swelling, and the sutures were removed. Follow-up at 6 months revealed no recurrence.

## 3. Discussion

Although facial cellulitis is most often caused by odontogenic infection, most commonly, untreated dental caries, periodontitis, or inflammation of the pericoronal tissues occur as a result of with semi-included teeth [6,7,9,10]. Facial cellulitis can also result from acute sinusitis, trauma with or without skin breakdown, foreign body reaction, external otitis, animal or insect bite, conjunctivitis, or blepharitis. All of these etiologies can be ruled out by the patient’s history and clinical examination. In addition, no mandibular fracture was found upon radiological examination. These findings, together with the patient’s symptoms, swelling, and trismus, indicate chronic infection and cellulitis due to odontogenic infection. The cause was probably related to the fracture of the tooth resulting from the punches he had received, or iatrogenically caused by the extraction of the second molar. Indeed, semi-impacted tooth number 48 presented a crack at the level of its mesial collar extended to the pulp chamber and in connection with the pericoronal space open on the alveolar crest, without periapical or periradicular lysis.

The symptoms of impacted teeth are often discreet, and their diagnosis is often late. However, certain clinical manifestations motivate a consultation. Indeed, an impacted tooth can be a source of numerous complications and the origin of the development of pathologies due to its close proximity to neighboring anatomical and dental structures [11]. Impacted teeth in children and adolescents are rarely associated with pathological changes, but the prevalence of problems tends to increase over the following decades. Therefore, careful and continuous monitoring must be established; this follow-up will include regular clinical and radiological checks and must be registered in order to detect possible complications and allow for the implementation of preventive or curative measures [12]. The most common complications are infectious and mainly affect impacted third molars because of the relationship between the follicular sac and the oral cavity.

Pericoronitis is accompanied by retro-molar pain and inflammatory signs such as congestive gingivitis, sometimes suppurative, and often lymphadenopathy. Pericoronitis can be complicated by acute cellulitis threatening the oropharyngeal junction and the freedom of the airways. The teeth of the anterior sectors are also affected, which can result in the following conditions: upper genital cellulitis circumscribed in canine palatal inclusions following untreated or poorly treated peri-coronary disease, thrombophlebitis of the facial vein which leads to thrombosis of the cavernous sinus, maxillary sinusitis in the event of an impaction, high ectopy or in the maxillary sinus, osteitis, and eye disorders [11,13,14,15,16].

Cystic or neoplastic lesions related to impacted teeth can also be a source of other infectious complications, which occur most often during the second and third decades of life [16]. The most common being dentigerous cysts [17]. They are observed at all ages, with a higher frequency in men between second and fourth decades in life. They mainly concern the third mandibular molar, the maxillary canine, and the second mandibular premolar.

Contemporary penitentiary systems pay significant attention, at least in terms of formal establishments, to providing inmates with appropriate health services and resocialization [18]. However, incarcerated persons are at increased risk of poor oral health compared to the general population. This increase in risk is linked to multiple factors [18,19]. It needs to be highlighted that inmates are exposed to greater access to illegal substances, such as drugs or nicotine. They often suffer from self-destructive patterns and personality or mental disorders. Prisoners are frequently diagnosed with anxiety, depression, bipolar disorder, psychosis, psychopathy, schizophrenia, and personality disorders [20]. Several studies have reported the oral health status of prison populations and have shown a high prevalence of dental caries, oro-mucosal lesions, precancerous lesions, poor periodontal status, and missing teeth [19,20,21,22,23]. Furthermore, violence in prison is generally frequent, with attacks between prisoners and towards staff, as well as resistance during arrest. This results in traumatic pathologies such as fractures or dislocations of the teeth and jaws [18]. Although the oral status of prisoners has been widely discussed in the available literature, none of the articles address the problem of wisdom teeth and related complications.

In our case, the cellulitis was related to an impacted wisdom tooth with pulpal necrosis in the context of a dental fracture caused by trauma. According to the patient, no extraction attempt had been performed by himself or a dentist as he had not consulted a dentist after the accident. To our knowledge, this appears to be the first case described in the literature of cellulitis of the face following an impacted wisdom tooth fracture. The fracture may have happened because the trauma affected a molar that was partly impacted. Surprisingly, the blows to the face had not resulted in a mandibular fracture.

### Learning Points

- This case describes unusual odontogenic infection of the face and subsequent management.
- In the case of accidents resulting in facial trauma, a practitioner should be consulted for a complete dental clinical examination.
- Damage to wisdom teeth should be considered as a possible cause of facial infection even if they have not erupted.
- The patient’s medical history is very important in resolving clinical cases.

## Data Availability

Data are contained within the article.
